# The Convention of the Illinois State Medical Society

**Published:** 1877-06

**Authors:** 


### Reports of Societies.

### THE CONVENTION OF THE ILLINOIS STATE MEDICAL SOCIETY.

The twenty-seventh anniversary meeting of the Illinois State Medical Society was held in the Grand Pacific Hotel on the 15th, 16th and 17th of May. On the morning of the first day, the delegates present were called to order at 10 A. M., by Dr. T. D. Fitch, of Chicago, the President of the Society, who requested Dr. J. H. Hollister to offer prayer.

The chairman of the Committee of Arrangements, Dr. James Nevins Hyde, of Chicago, then made a brief address of welcome, and announced the hours of meeting, the order of business, and several invitations to visit the medical and scientific institutions of the city. Dr. J. L. White, of Bloomington, responded to Dr. Hyde's words of welcome, in appropriate terms.

After some time, which was expended in arranging the hours of hearing reports and papers, the President delivered the annual address, of which the following is a brief synopsis:

He hoped that harmony would prevail during the present session, and that it might close with the feeling on the part of all who had attended that it had been a place where it was good to be. He referred to the advantages arising from organizations as a general thing, and thus educed the advantages which accrued from a society like the present. He thought it the duty of every medical man to connect himself with a medical society. He urged that valedictorians should especially impress this, and showed that even those men who in prosperity ignored societies, in time of trouble and in need of protection, recognized the advantages of them. He showed the good to be derived from societies in matters of mutual protection, in interchange of knowledge and experience. Much could be gained by connection with such a society, and many problems could be solved by joint labors therein, which could not by individual endeavor. He considered it the duty of every physician to take all means to make himself worthy of his high calling, and, referring to the young students, as a class, declared that scientific knowledge could nowhere be better obtained than by associating with other members of the profession. Reciprocal advantages also accrue both to the young and the old practitioners, the former acquiring the practical knowledge of the latter, and the latter securing the theoretical knowledge of the former. He considered that every medical man in the State should feel it incumbent upon himself to come up to the annual feast of medical knowledge. He referred at some length to the society, its origin, the superior character of its early members, the firm basis upon which the organization had been founded. The social advantages to be derived from such a society were invaluable and incalculable, it being the nature of man to seek fellowship and derive benefit therefrom. He considered the present mode of receiving members pernicious. Many persons were elected who never attended the meetings; who were appointed to committees which never made reports; who never paid their dues—which he considered a sacred duty. He maintained that the membership of any person behind in his dues should not be perpetuated beyond two years. The relations of the society towards other similar societies were amicable and pleasant, and the society was treated with deference everywhere. The mechanical management of the society he considered excellent. He referred to the innovation which had been introduced by the appointment of Mrs. Sarah Hackett Stevenson as a delegate to the American Medical Association at Philadelphia, which had been brought about by himself and the late Dr. Freer. He considered that women, whenever competent, should be admitted to membership and representation, and considered that this society had cause to congratulate itself upon having sent the first lady delegate to the general association.

But the angel of death had not stayed its hand during the year; and among those of the society whom it had snatched away, he wished to mention Dr. J. W. Freer, and Dr. G. G. Goll, both of this city. With the former he had had personal acquaintance, but, sparing his own words, would read the obituary notice which appeared shortly after the death of the departed, and gave a somewhat extended résumé of the life of the departed. He declared the deceased to have been a man of clear judgment, noble and upright in his private and professional life. Such men add dignity to the profession which they represent.

Dr. Fitch read the resolutions which were passed relative to the death of Dr. Freer by the medical profession of the city of Chicago.

In referring to Dr. Goll, the speaker declared him to have been a young man of fair talent and bright prospects.

He concluded with thanking the society for the honor conferred upon him in being appointed to the Presidency, and expressed the hope that the lamp of the organization should continue to burn.

Upon motion of Dr. E. Ingals, it was decided that the President's address should be accepted and published.

After the transaction of unimportant routine business, the meeting adjourned till half-past 2 o'clock.

At 2½ P. M., Dr. E. W. Gray, of Bloomington, presented the Report of the Committee on the State Board of Health, together with the petition for the establishment of the same, and the bill as originally prepared. The amendments made in the bill, both as to the powers of the proposed board and the appropriaion asked for, were also stated. The report was accepted and referred for publication.

Dr. N. S. Read, of Chandlerville, Chairman of the Committee on Practical Medicine, then presented his report, Dr. E. F. Ingals also reading a supplementary paper.

Dr. E. Andrews, of Chicago, then, by permission, exhibited a patient, a little girl eight years of age, affected with empyema, in whose case paracentesis had been performed. By attaching a tube of a fountain syringe to the drainage tube inserted, the left thoracic cavity was thoroughly washed out with a carbolated solution.

Dr. I. P. Walker, of Mason County, read a short paper, following this, in connection with the Report on Practical Medicine. The discussion of the entire subject was conducted by Drs. C. M. Fitch, D. S. Jenks, J. S. Whitmire, J. S. Jewell and others.

Dr. J. L. White, of Bloomington, then read the Report on Surgery, of which the following is a synopsis:

The Chairman of this Section confined himself to concise reports of somewhat unusual cases occurring in his own practice and that of neighboring surgeons, and comments of a practical nature, upon the same. He first reported a case of dislocation of the femur, of eleven weeks standing successfully reduced. Also, two cases alike successful, of dislocation of the humerus, of two years duration. From these cases, was urged the importance and propriety of attempting the reduction of dislocations generally, after a much longer time has elapsed than is laid down in standard surgical works as justifiable. He then gave the history of some cases known to him, in which there was in some complete, and in some partial, anchylosis of the elbow joint, going to show the superior advantages of a nearly straight position of the arm over one bent at right angles, and urged the importance of considering the avocation of a patient in dressing the injuries of this joint, when anchylosis was liable to result. A case of severe injury to hand and arm was reported, in which good results were obtained, by keeping the parts for some weeks submerged in hot water, and the advantages of this method of treatment, illustrated and urged upon the profession. The question of gastrotomy was discussed in reference to cases of intussusception, and cases remarked upon where this had been successful. Also some general remarks upon the comparative safety of opening into the peritoneal cavity, taking the ground that the fatal results following this operation have generally been the result of septicæmic poisoning rather than tissue inflammation.

He reported cases of embolism, in which limbs had died from the plugging of the femoral artery, and suggested the importance of great care in the changing of much enfeebled persons from a recumbent to an erect position. The question of fractures was discussed, and the almost inevitable consequent shortening insisted upon, taking the ground that any unusual attempt to prevent this by excessive extension was liable to be followed by non-union of the fractured bone. In this connection, the profession were reminded of their duty to one another, in regard to giving currency to the idea that no shortening or deformity ought to be the result of fractures. The general use of patent splints and appliances was rather discouraged than recommended. A very interesting and instructive case was reported of death resultiug from the use of Esmarch's bandage, and the necessity for the employment of the greatest care and caution in its use, insisted upon. The fatal result in this case was apparently due to the powerful impression made upon the cutaneous nerves, amounting to paralysis. Cases were reported, showing a condition of the system frequently to exist, in which there was no disposition for a wound or injury to heal, cases in which the blood seems to be devoid of its normal plastic properties, and for the relief of which iron is demanded, and in which its use should be perseveringly and persistently employed. The importance of antiseptics in all surgical dressings was insisted upon, and the free exit to all purulent accumulations, in contact with absorbing surfaces. In this connection, the question of dressing stumps, after amputation open, without either sutures or adhesive straps was discussed, and in a general way, approved, though not distinctly recommended. The great superiority of ether over chloroform, on account of its comparative safety, was urged, and the hope expressed that some safe agent might be discovered with which it could be combined, rendering it more prompt in its action, and more easy of administration. Torsion of arteries was claimed to be far preferable to any ligature, when appropriate, and it was insisted that in a great majority of instances it would accomplish all that was required. A photograph, exhibiting the practical working of skin grafting was shown in connection with the report of a case, where great benefit was derived from its employment.

On the morning of the second day, Dr. C. T. Wilbur, of Jacksonville, read an interestiug paper on Idiocy. The author said that nearly or quite one-third of the patients admitted to the asylum at Jacksonville, in the course of a very few years were so far recovered as to be permitted to go at liberty and care for themselves. Many, of course, were hopelessly incurable, there being no basis to work on in consequence of mental deficiency. The proportion of the whole number of patients admitted to the institution who were incurable was quite small. It was his opinion that idiots should be treated apart from other patients. They should be kept excluded from lunatics and paupers, as the disturbing influences which surrounded these persons were very harmful. The simpler the lesson and the kindlier the treatment of these patients, the sooner good results will be developed. A thorough industrial training was also indispensable. The work should be light, and never forced upon the patient. The author thought there was as much difference between the trained and untrained idiot, as between the civilized man and the savage. Idiots required at all times kind, gentle treatment, no matter what might be their actions. They were as helpless, mentally, as children, and the same treatment given the child was requisite for the feeble-minded.

The report was received.

Dr. Plym. S. Hayes then read a report on Electro-Therapeutics, setting forth his own experiments with various instruments, and giving detaiis of several cases in which the therapeutic value of this agency was demonstrable.

Dr. E. M. Griffith, of Springfield, next presented the Report of the Committee of Drugs and Medicines. He laid special stress on the necessity to the profession of great care in the use of medicines, not alone for the preservation of their own reputation but the health, and perhaps life, of their patients. There was no legislation in Illinois preventing quackery. This was unfortunate. The manufacture of inferior and worthless medicines was entirely too common, and a stop should be put to it. There should be some means of preventing ignorant and unskilled men from flooding the market with spurious nostrums. The history of drugs, and the uses and properties of many, were carefully and intelligently reviewed, and the metric system of weights condemned as worthless in the measurement of drugs.

The report was referred to the committee on publication.

Dr. N. S. Davis denounced the practice of countenancing the use of proprietary medicines and the foisting upon the profession of ready-made, worthless, impure prescriptions. So great was his antipathy to patent-medicine men, and to the agents of those popular remedies, that he declared he had refused in his medical journal to advertise such medicines. All that the profession wanted was, that the pharmacist should fill its prescriptions, and not seek to impose on it these ready-made prescriptions. The druggist and the pharmacist had a standard that the profession agreed to as the officinal standard of strength and purity of drugs, and every druggist that dispenses medicine should be held responsible for dispensing it according to that standard, and every physician should direct exactly what combinations he wanted for his individual patients. He thought, in conclusion, it would be a good plan to have the Committee on Drugs and Medicines institute experimental inquiries into the nature of existing drugs, with special reference to pruning the materia medica, or at least of reducing the present vague and indefinite knowledge on this subject to a more exact appreciation of what each medicine could do.

Dr. Jewell said that there was still no scientific method for the treatment of hydrophobia, although it had been confidently hoped of late that one was about to be discovered. The doctor called attention to the importance of the subject of the therapeutics of Rest and Position, and made a promise to contribute something on this subject at the next annual meeting.

Dr. A. Hard, of Aurora, spoke of oxygen gas as valuable in therapeutics.

Dr. J. S. Whitmire, of Metamora, related a case that had been reported to him where the excessive use of chloral to produce sleep had rendered the persons who used it insane. Opium was substituted for a time, and a recovery was apparent in three or four weeks.

Dr. W. L. Ransom, of Roscoe, another member of the Committee on Drugs and Medicine, read an interesting paper on Apocynum Cannabinum, and directed special attention to its value as a diuretic.

Dr. N. S. Davis followed with some remarks on the same subject. He had found the Apocynum Cannabinum a valuable remedy in dropsy from diseases of the kidneys. It acted as a sedative, and, when properly used, was exceedingly valuable as a remedy in all such diseases.

Dr. Johnson, of this city, spoke of the successful employment of Indian Hemp in Bright's disease of the kidneys.

Dr. Bartlett said the root alone of the Indian Hemp was efficacious.

Dr. J. L. White, of the Board of Censors, reported the case of Dr. Barnett, who had made and subsequently withdrawn certain charges against the Macoupin County Society. It appeared from the evidence that he had associated himself with an irregular physician in that county. The County Society expelled the said Barnett, and the latter preferred charges against the Society. The Board of Censors was of the opinion that expulsion from a local society should work expulsion from the State Medical Society. The Board recommended the admission of the following applicants for membership: Drs. Ransom Dexter, S. O. Richey, T. J. Bluthardt, W. Godfrey Dyas, A. H. Foster, Chicago; C. H. Lovewell, Englewood; C. S. Taylor, Rockton; C. N. Cooper, Batavia. The report was adopted.

A report was made by Dr. Charles W. Earle, from the committee on Diseases of Children.

The burden of the report referred to the scarlatina epidemic prevalent in Chicago during 1876-'7. Speaking of the early mortality from scarlatina, he stated that the disease had been epidemic in this city since 1851, the years 1858, '59, '62, '63, and '69 being marked by severe epidemics. The doctor said the epidemic which raged last October had not been as severe as malignant as had been represented. He did not think there was any lack of material to form a malignant epidemic, and with the utter indifference of the profession and the want of knowledge among the people of the alarming contagiousness of the disease, with the daily papers filled with communications promising immunity if certain medicines were used, he only wondered that our mortality had not been by thousands instead of by hundreds.

Taking as a basis for calculation nearly one hundred reports he had received from physicians, he estimated the entire number of scarlatinous cases as 14,170 during the last sixteen months—the number of deaths being 1,191, or 8½ per cent. Any epidemic with a mortality of less than 10 per cent. was considered very mild, and he cited several epidemics in other countries with 25, 30, and one with a death-rate of 40 per cent.

Tables showing the number of children under the age of sixteen years were given in each waid of the city. In the Fourteenth ward, there are over twenty thousand, and in the entire city, over one hundred and fifty-two thousand, sixty-three thousand of whom were in public and private schools.

At the opening of the schools last year, it was estimated that there were one hundred and fifty infected points. Notwithstanding the favorable condition for the spread of the disease in the schools, only one in every one hundred and twenty-eight persons had died from the disease.

The doctor discussed each ward at some length, giving the sanitary condition of each, deaths from the disease, proportion to population, etc.

In conclusion the speaker said a report of the kind he presented would not be complete without saying something in regard to preventive medicine. Belladonna was discussed fully, both the claims for and against its use being given. He concluded that the general experience in the use of this article had not increased its reputation. The history of carbolate of sodium was given, and the results of its use in the city, presented. In some ninety cases where it had been given to prevent the disease, forty-eight had been affected with scarlet fever.

The report was received, and the society tendered its thanks to the reporter.

The name of L. R. Williams was proposed for membership and referred.

The society then took a recess until 2:30 P. M.

At 2:30 o'clock the convention reassembled in the club-room adjoining the rotunda, and the transaction of business was resumed.

The president called the meeting to order, and asked for the report of the nominating committee. In response, Dr. Earle read the names which were approved.

Dr. J. H. Hollister then read a report on Dietetics and Hygiene of Children.

The doctor divided the report as follows: The subject of infant and childhood mortality. The causes which conspire to this result; viz., Paternal influences, congenital malformations and weaknesses, unhealthy surroundings, contagious diseases, improper clothing and erroneous diet. General observations upon diet, with a variety of formulæ where alien food is needed. Nursery rules for the care and feeding of infants.

The author of the paper said it was with the first of these points he would mainly have to do. Infancy, as the term implies, is that period in which a human being is without a language, and though is may not yet be able to articulate its mother tongue, it has a language that no human heart can fail to understand. It is truly the period of utter helplessness, and yet it has the power to command the purest and the strongest of human agencies. This period of infancy, the period of most delicate development and the embodiment of weakness, is, nevertheless, the period of most intense vital activities and physical changes, and it is during this period that life is imperiled as in no other, and thus we find that one-tenth of the human race die before they are one month old, and more than one-half of the human family before the age of five years. The tables of both England and the Continent confirm this fact as regards the old world, and the same is true of this country as well. We are not able to give as exact data for the country at large, but in the populous cities, where statistics are complete and reliable, the same fact obtains. For example, in New York city, 26 per cent. of the deaths are those of children under one year old, and 53 per cent. die less than five years old.

In Chicago the statistics are as follows:

| | |
|---|---|
| Total number of deaths reported during the year 1876, | 8,573 |
| Died under one year, | 2,694 |
| Those under two years, | 1,038 |
| Those under three years, | 518 |
| Those under four years, | 396 |
| Those under five years, | 245 |
| Total, under five years, | 4,891 |

The per cent. of the entire mortality being 57.

The speaker said these figures were enough to appall the strongest heart. They not only point to the untold sufferings that went out with these lives, from pain endured, but they remind us of the aching hearts and beclouded homes which have been on every side of us during the past year. The voices of philanthropy and of pity not only ask, "Must this be?" but the economist inquires as well.

These reflections led the speaker to the consideration of the causes which produce such fearful fatality. Some of the cases were obviously beyond the agency of human skill, and while they swell the death rate are not amenable to treatment. Such, for example, are the congenital malformations of infants; such as hydrocephalus and acephalous affections, spina bifida, cyanosis, cardiac, and pulmonary lesions; malformations of the secretory and digestive organs, and failures of development of almost an endless variety.

Hereditary taints are apt to manifest themselves early, and many times these baffle the best directed efforts.

Inherent weakness at birth is a most fruitful sourse of fatality. The feeble development of the organism is often entirely unequal to the wants of life, and in the unequal struggle it yields that life. A like result of feebleness inheres from the very extremes of human condition.

The doctor reviewed the causes of disease and death in children, dwelling upon the necessity of subsisting children on food of a proper character, holding that nature's food was the only one which should be used in every instance practicable.

Dr. Matthews then read a paper on the subject of reflex phenomena induced by congenital phimosis, illustrated by several cases.

Both Dr. Hollister and Dr. Matthew's reports were received.

A general discussion then ensued on Dr. Earle's report, and those of Drs. Hollister and Matthews.

The committee on nominations retired for the transaction of business.

On motion, a committee of three was appointed to prepare a circular for general circulation, containing suggestions on dietetics and hygiene of children.

Dr. J. L. Hayes, of Paris, presented a report on ophthalmology.

Dr. Jno. Wright presented a paper on Hæmorrhoids, illustrated by cases.

Dr. E. Andrews, of Chicago, then read a paper, setting forth a new method of producing extension and counter-extension in diseases of the ankle-joint, by means of a leathern and laced splint, covering the dorsum of the foot and reaching over the calf of the leg. The principle of the apparatus was simply equal compression of two cones joined at their apices. The paper was illustrated by a plaster model, with the apparatus *in situ*.

Prof. E. L. Holmes read a paper on Foreign Bodies in the Globe of the Eye.

Dr. Richey followed with a paper on Diseases of the Middle Ear in Connection with Nasal and Pharyngeal Inflammation.

The Society then adjourned.

On the morning of the 3d day, Dr. J. Adams Allen, Chairman of the Committee on Necrology, read the report of that committee, including a paper on the career and labors of the late Dr. Freer, of this city.

At the request of the Convention, Dr. Allen also read a report of the autopsy in Dr. Freer's case, which, with the committee report, was referred to the Committee on Publication.

Dr. Worrell asked leave to make a slight contribution to the necrological report, and the same was, on motion, added.

The order of exercises was, on motion, suspended. The Board of Censors reported favorably to the election of the following gentlemen to membership: Dr. H. Hatch, New Salem; Dr. G. E. Willard, Chicago; Dr. J. H. Bates, Chicago; Dr. L. R. Williams, Chicago. The Board also made the following request: "There being no evidence to sustain charges preferred against the Jersey county Medical Society, we would recommend that the charges be dismissed." The report of the Board was adopted.

The subject of the establishment of a State Medical Institute was presented and discussed at some length. Dr. John H. Hollister, Treasurer of the Society, reported as follows: Balance on hand per last statement, $10.82; cash received at Champaign, $331; cash collected during the year, $222; cash advanced by the treasurer, $62.73; total, $626.55. The disbursements for the year were $626.55 for various objects of the Society. On motion, the report was audited and approved.

The Committee of Publication reported through Dr. N. S. Davis, and the report was accepted.

Dr. Cook, of Mendota, from the Nominating Committee reported the following nominations of office for the ensuing year:

*President*—J. L. White, of Bloomington.

*First Vice-President*--E. P. Cook, Mendota.

*Second Vice-President*—B. M. Griffith, Springfield.

*Treasurer*—John H. Hollister, Chicago.

*Assistant Secretary*—H. B. Buck, Springfield.

*Permanent Committees*—Board of Censors—M. W. Walton, Ridott; E. Ingals, Chicago; and P. H. Barton, Danville. *Practical Medicine*—C. W. Earle, Chicago; D. S. Booth, Sparta; and J. T. Pitner, Jacksonville. *Surgery*— Moses Gunn, Chicago; W. P. Pierce, Lemont; and David Prince, Jacksonville. *Obstetrics*—T. D. Fitch, Chicago; E. R.

Willard, Wilmington; and Ellen Ingersoll, Canton. *Drugs and Medicines*—N. S. Davis, Chicago; E. P. Cook, Mendota; and William E. Quine, Chicago; *Diseases of Children*—C. C. Hunt, Dixon; Harriet Botsford, Chicago; and Lucinda Corr, Carlinville. *Neurology*—H. A. Johnson, Chicago; T. F. Worrell, Bloomington; and Thomas Gault, Rock Island.

*Special Committees—Otology*—Samuel J. Jones, Chicago. *Neurology*—J. S. Jewell, Chicago. *Ophthalmology*—E. L. Holmes, Chicago. *Electro-Therapeutics*, P. S. Hayes, Chicago; *Malaria*, N. Wright, Chatham. *Fractures of Lower Extremities*—W. L. Goodell, Effingham. *Croup*—H. Z. Gill, Jerseyville. *Throat and Nasal Passages*—J. P. Matthews, Carlinville. *Dysentery*—John Wright, Clinton. *The Chronic and Incurable Insane Poor of Illinois*—R. J. Patterson, Batavia.

Springfield was decided upon as the place of holding the next Convention, and Drs. B. M. Griffith, J. Townsend, and A. A. Patterson, all of that city, were appointed a committee of arrangement. The report was adopted.

Dr. J. S. Jewell, of Chicago, addressed the Convention upon the subject of "The Influence of the Nerves Over Nutrition." Dr. Jewell had spent two years in the investigation of this particular subject, and his paper was appreciated by those present in a vote of thanks.

Dr. S. J. Jones read a paper upon the diseases of the eye and ear, and was thanked by the Convention.

The Convention then passed a vote of thanks to the Committee of Arrangements, the proprietors of the Grand Pacific Hotel, to the local medical societies and members of the profession for entertainment, and to the press.

On motion, Drs. N. S. Davis, E. P. Cook, T. D. Fitch, and Bogue were appointed a committee to revise the constitution and by-laws of the Society.

Dr. C. W. Earle read a preamble and resolution, which recommended that the Chicago Medico-Historical Society be authorized to publish an annual Medical Register of the State of Illinois, agreeably to a resolution of the American Medical

Association. Adopted. Some additional communications were read and accepted.

Delegates were appointed to represent the Society at the several State Conventions next year, and twenty-eight delegates were elected to the American Association for 1878. The retiring President, Dr. T. D. Fitch, then thanked the Convention for the honors conferred upon him and for their attention and courtesy under his Presidency. The Society then adjourned, to meet one year hence at Springfield.

On Wednesday evening the Medical Profession of the city of Chicago, through the agency of the Chicago Medical Society and the Chicago Society of Physicians and Surgeons, entertained the visiting members of the State Society in a banquet at the Grand Pacific hotel.

